# Effects of exercise with or without a hypocaloric diet on intermuscular and intramuscular fat: a systematic review

**DOI:** 10.1007/s40520-025-03097-2

**Published:** 2025-06-09

**Authors:** Konstantinos Prokopidis, Alyssa N. Varanoske, Nicola Veronese, Ben Kirk, Konstantinos Katsikas Triantafyllidis, Christoforos D. Giannaki, Pinelopi S. Stavrinou, David D. Church, Gustavo Duque

**Affiliations:** 1 Department of Musculoskeletal and Ageing Science, Institute of Life Course and Medical Sciences, University of Liverpool, Liverpool, UK; 2 KBR, Exercise Physiology and Countermeasures Laboratory, NASA Johnson Space Center, Houston, TX USA; 3 Geriatric Unit, Department of Internal Medicine and Geriatrics, University of Palermo, Via del Vespro, 141, 90127 Palermo, Italy; 4 Department of Medicine, Melbourne Medical School, Western Health, University of Melbourne, Melbourne, VIC Australia; 5 Australian Institute for Musculoskeletal Science (AIMSS), University of Melbourne and Western Health, Melbourne, VIC Australia; 6 Department of Nutrition and Dietetics, Royal Marsden NHS Foundation Trust, London, UK; 7 Department of Life Sciences, School of Life and Health Sciences, University of Nicosia, Nicosia, Cyprus; 8 Department of Geriatrics, Donald W. Reynolds Institute on Aging, Center for Translational Research in Aging and Longevity, University of Arkansas for Medical Sciences, Little Rock, AR USA; 9 Bone, Muscle & Geroscience Group, Research Institute of the McGill University Health Centre, Montreal, QC Canada; 10 Dr. Joseph Kaufmann Chair in Geriatric Medicine, Department of Medicine, McGill University, Montreal, QC Canada

**Keywords:** Exercise, Intermuscular fat, Intramuscular fat, Adipose tissue, Hypocaloric diet

## Abstract

**Background:**

Intermuscular (InterMAT) and intramuscular (IntraMAT) adipose tissues are key contributors to skeletal muscle function and metabolic health.

**Objective:**

In this systematic review, we aimed to investigate the impact of different types of exercise with or without a hypocaloric diet on reducing InterMAT and IntraMAT in various muscle tissues.

**Method:**

A literature search was performed via PubMed, Cochrane Library, Scopus, and Web of Science from inception until February 2025. Eligible RCTs examined the effects of exercise compared to control in adults > 18 years. A narrative synthesis was employed with a registered protocol at CRD42024511531.

**Results:**

Nineteen RCTs were included in the systematic review. Resistance and aerobic exercise alongside a hypocaloric diet displayed inconsistent results in reducing thigh InterMAT. Under non-hypocaloric conditions there were consistently no alterations of InterMAT and IntraMAT irrespective of muscle tissue. When concurrent exercise was followed, no statistically significant changes were observed with or without a hypocaloric diet on thigh InterMAT and IntraMAT. Results were overall lacking in regards to resistance or aerobic exercise and IntraMAT due to the low number of studies.

**Conclusion:**

There is potential in reducing thigh InterMAT following resistance or aerobic exercise combined with a hypocaloric diet, although inconsistencies among studies were presented. However, irrespective of exercise type, under non-hypocaloric conditions no benefits were conferred on InterMAT and IntraMAT of any muscle tissue. Further research is warranted to determine the effects of different exercise intensities employing an adequate dietary and hypocaloric control on skeletal muscle InterMAT and particularly IntraMAT.

**Supplementary Information:**

The online version contains supplementary material available at 10.1007/s40520-025-03097-2.

## Introduction

Physical activity and exercise have been recognized as essential components of a healthy lifestyle, providing benefits to cardiovascular health [1], muscle mass, strength, and physical performance [2] that correspond to better quality of life and reduced mortality [3, 4]. Some of these benefits may potentially be explained in part by changes to intramuscular adipose tissue localized within skeletal muscle fibres (IntraMAT), and intermuscular adipose tissue localised between distinct muscle groups (InterMAT). Both IntraMAT and InterMAT possess key roles in metabolic health, for which, their reduction through exercise has become a subject of growing interest in the fields of exercise physiology and obesity research. In particular, IntraMAT and InterMAT content have been shown to decrease with prolonged physical activity [5–8] or its combination with dietary restriction [9]. Increases in both tissues may reduce skeletal muscle insulin sensitivity, potentially through the secretion of inflammatory cytokines and extracellular matrix proteins, as well as by elevating local concentration of free fatty acids (FFA) [10, 11]. Furthermore, as adipose tissue plays also a key role in muscle function, both IntraMAT and InterMAT accumulation have been found to be associated with poor physical performance [12], such as gait-speed decline [13], and reduced muscle strength [14]. Accordingly, their decrement through exercise and diet was associated with improvements in physical function [7, 15].

The type of exercise may play a pivotal role in determining the extent of changes in IntraMAT and InterMAT, however, little is known around this area. Aerobic exercise, resistance training, and a combination of both may influence adipose tissue distribution within and around muscles [16], although, such changes may not correspond equally to different muscle groups [17]. Some of these changes may underpin the metabolic impact that each type of exercise, to different extents, could exert on reducing inflammation, assisting with body fat losses, and improving insulin sensitivity [18, 19]. However, considering the multifactorial nature of weight loss and subsequent reductions in different adipose tissues, evidence is lacking in support of aerobic or resistance training per se. Furthermore, these exercise-induced adaptations highlight the dynamic nature of adipose tissue and highlight the importance of tailoring exercise prescriptions for individuals with specific health goals. Moreover, dietary interventions targeting weight loss may also potentiate improvements in metabolic health by further reducing myosteatosis in skeletal muscle independent of changes in subcutaneous or visceral fat [20]. Previous studies have demonstrated favourable effects of exercise in alleviating these adipose tissues [21–24], however, the relationship appears to be complex and may vary depending on factors such as exercise type (e.g., resistance training, aerobic exercise), intensity and duration, specific muscle tissue from which InterMAT and IntraMAT were measured from, as well as the dietary protocol followed (i.e., following a weight-loss intervention or controlling for dietary intake).

As mentioned above, there is evidence indicating that various forms of exercise can reduce different types of adipose tissue present in skeletal muscle. However, it is unclear whether this is achievable in cases where the individual does not concurrently follow a diet, such as a hypocaloric diet, or which form of exercise is most effective when combined with a diet. For example, in the study by Waters et al. (2022) on older adults with obesity, the results showed that the most effective intervention for reducing InterMAT and other metabolic markers was the combination of aerobic and strength training along with a hypocaloric diet, compared to groups that performed only aerobic exercise, only strength training, or no exercise at all [19].

Unravelling the specific effects of exercise on InterMAT and IntraMAT and its metabolic consequences is an ongoing area of investigation with implications for the management of sarcopenia and/or obesity. Characteristics of exercise (strength, HIIT, submaximal, concurrent) are factors influencing muscle growth/protein synthesis, energy expenditure, and finally muscle composition [25, 26]. The limitation of sarcopenia (elderly population) and/or the benefit in weight loss (obesity) seems to depend on the characteristics of the exercise. In this systematic review, by taking into account critical confounding factors such as dietary patterns and type of exercise, we attempt to explore the underlying exercise-induced changes in InterMAT and IntraMAT, aiming to provide valuable insights that could inform clinical practice and public health guidelines on the efficacy of exercise interventions on mitigating fat infiltration in and around the skeletal muscle.

## Methods

This systematic review was conducted based on the Preferred Reporting Items for Systematic Reviews and Meta-Analyses (PRISMA) guidelines, with a protocol registered in the International Prospective Register of Systematic Reviews (PROSPERO) (CRD42024511531) [27].

### Search strategy

Two independent reviewers (KP, KKT) searched PubMed, Scopus, Web of Science, and Cochrane Library from inception until February 2025. Table S1 describes the full search strategy and the search terms used in each database. A third reviewer resolved discrepancies in the literature search process (NV).

### PICOS criteria

Studies met the inclusion criteria if:

#### Population

i) they included participants with a mean age above 18 years old irrespective of health status.

#### Intervention

ii) studies followed an exercise protocol of any type and frequency and described/followed some type of dietary intake control during and/or around the study (i.e., baseline and during follow-up)/were provided dietary supervision from authors or a healthcare professional, such as a registered nutritionist or dietitian. Study duration should have been 4 weeks minimum.

#### Comparator

In case a hypocaloric protocol or a pharmacological agent was being administered in the intervention group, the comparator group should also have had an identical protocol in order to distinguish the (additional) impact of exercise on different adipose tissues in or around the muscle.

#### Outcomes

Studies measured IntraMAT and/or InterMAT from any muscle group using magnetic resonance imaging (MRI) or computed tomography (CT).

#### Study design

Randomized controlled trials (RCTs).

Studies were excluded if:

They were (i) published dissertations or conference abstracts; (ii) included individuals below 18 years of age; (iii) included an active control outside of usual care; (iv) in vitro and animal studies; and (v) studies were not written as full-text or in English. A PICOS principles table is presented in Table S2.

### Data extraction and risk of bias

Two reviewers (KP, KKT) extracted data independently, including the name of the first author, date of publication, number, mean age, body mass index (BMI), health status, and sex of the participants, outcomes of interest, exercise type (aerobic or resistance, or both), intensity, frequency and duration, hypocaloric protocol and how it was controlled, InterMAT and IntraMAT content, and skeletal muscle fat infiltration assessment tool. Disagreements between authors were resolved by a third independent reviewer (NV). Data presented only in figures or graphs were obtained using image analysis software (Image J, version 1.52a, National Institutes of Health, Bethesda, MD, USA) by digitally measuring the height of data points and error bars and calculating relative to measured y-axis units. Quality of included studies was evaluated via the risk-of‐bias 2 (RoB2) tool [28] by two reviewers (KKT, KSK) and any discrepancies were resolved by another independent member (KP). RoB2 is used to assess bias in RCTs based on the following domains: (i) randomization process; (ii) deviations from intended interventions; (iii) missing outcome data; (iv) measurement of the outcome; (v) selection of the reported result. Based on the score, bias was defined as ‘high’, ‘some concerns’, or ‘low’ [29].

### Data synthesis

Quantitative data were treated as continuous measurements and any standard errors (SE) were transformed to standard deviations (SD), using the formula SE = SD/√n or SE= (Upper CI limit − Lower CI limit/2 × 1.96) to improve the consistency across studies that would assist with the interpretation of the systematic review *via* a narrative synthesis.

## Results

### Search results

**Fig. 1:**
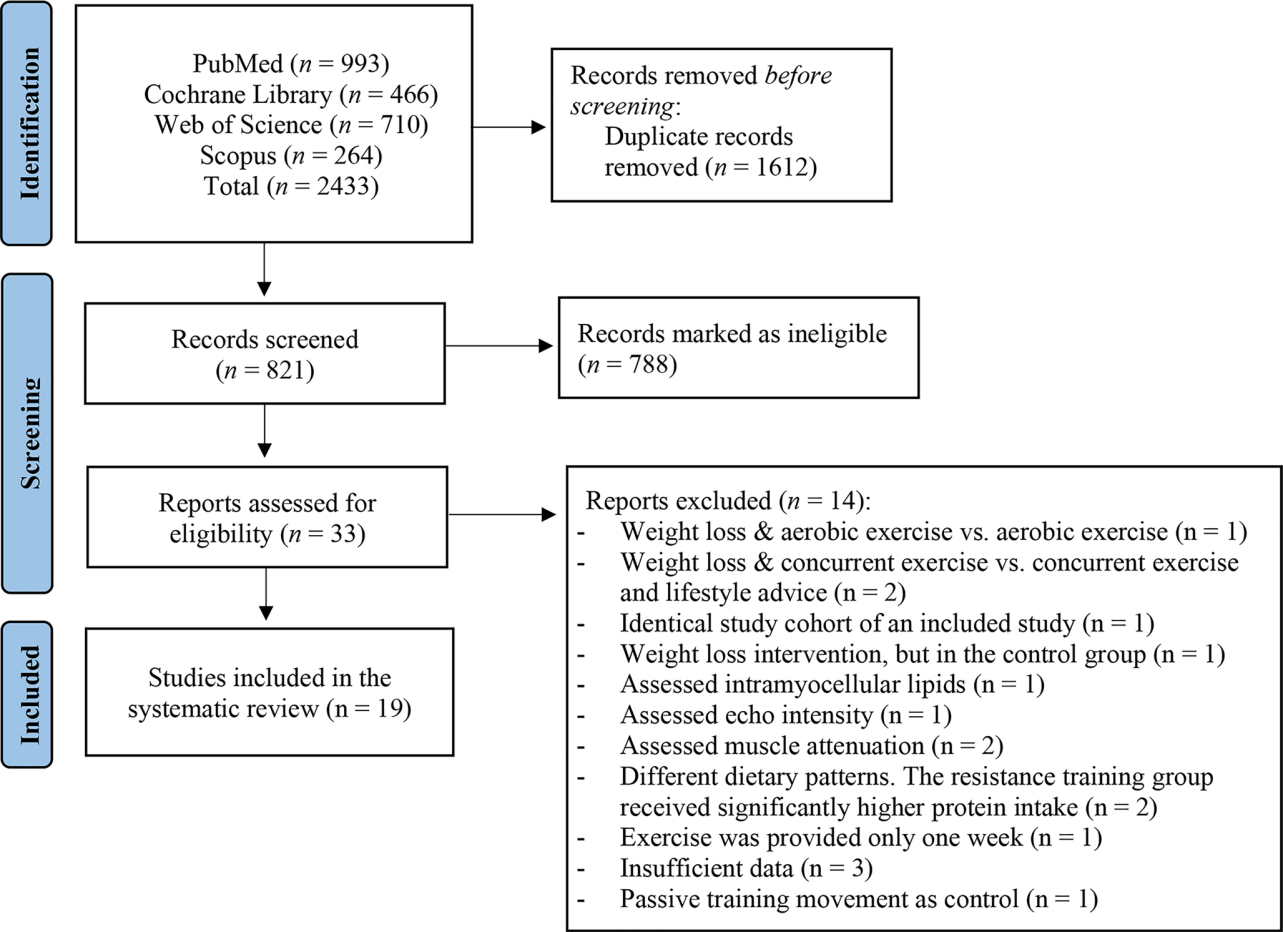
Flowchart of the search strategy employed in the literature search

The initial literature search displayed 2433 publications. Following the exclusion of 1612 duplicates, 821 unique publications were screened. In total, 788 publications were marked as ineligible, while full-text screening of the remaining 33 publications resulted in 19 eligible RCTs examining the impact of exercise with or without a hypocaloric diet on IntraMAT and InterMAT (Fig. 1). More specifically, 12 studies incorporated exercise combined with a hypocaloric diet [19, 30–40], while seven studies incorporated exercise without a hypocaloric diet [41–47]. The majority of the eligible studies combining hypocaloric diet and exercise involved overweight/obese older adults, while in the studies analysing exercise without hypocaloric diet, both healthy status and age show greater heterogeneity. A detailed table of characteristics of the included studies is presented in Tables 1 and 2.

**Table 1:** Study and participant characteristics of the included studies using a hypocaloric dietary protocol. Data are expressed as mean (standard deviation) or range

Study, year	Health status	Outcome of interest	Intervention			Control			Training protocol	Duration	Assessment tool	Dietary intake control
			*n* (M/F)	Age (SD)	BMI (kg/m^2^)	*n* (M/F)	Age (SD)	BMI (kg/m^2^)				
Avila et al., 2010	Overweight & Obesity	Thigh InterMAT	9/6	66.0 (3.8)	31.6 (3.8)	7/5	67.4 (4.8)	31.9 (3.4)	3x/week; 40 min of moderate intensity; 4 sets of 8–12 reps for 6 exercises/session	10 weeks	CT	Only at baseline; DASH diet; All subjects were given an individualized diet at the start of the intervention for a 10% calorie reduction. All diet sessions were led by a dietitian.
Brennan et al., 2022	Obesity	Thigh InterMAT	8/12	66.8 (3.4)	37.3 (5.4)	7/14	70.0 (4.6)	36.1 (5.1)	4–5x/wk, 45 min per session; walking (outside and on an indoor treadmill) and the option to include stationary cycling, elliptical, and rowing machines; At Week 8, participants were also prescribed 2 non-consecutive resistance exercise sessions/wk, 30 min/d, (2–3 sets of 10–12 reps)	6 months	MRI	A registered dietitian ensured a 500–1000 kcal reduction/day
Madrid et al., 2023	Obesity	Thigh InterMAT	7/12	Aerobic: 65.8 (5.1); Resistance: 65.0 (3.8)	Aerobic: 33.6 (3.5); Resistance: 34.1 (3.7)	6/11	66.8 (3.9)	34.4 (3.6)	Aerobic: 4 x/wk; walking for 45 min/d; 12–14 Borg Scale– Resistance: 45 min/d with 3 sets of 10–12 at 75%1RM upper and lower body machine-based exercises	18 months	CT	Caloric deficit of about 330 kcal/d for months 1–6; months 7–12 were about gradually going towards maintenance and months 13–18 was maintenance
Nicklas et al., 2015	Overweight & Obesity	Thigh InterMAT	26/37	69.6 (3.9)	30.4 (2.2)	29/34	69.4 (3.6)	30.7 (2.4)	3d/wk moderate intensity resistance exercise; 3 sets of 10 reps at 70%1RM	5 months	CT	Participants were asked to keep a diet log of all foods consumed, and the logs were monitored weekly by a registered dietitian to verify compliance with the weight-loss intervention
Waters et al., 2022	Obesity	Thigh InterMAT	Aerobic: 14/26; Resistance: 15/25; Concurrent: 16/24	Aerobic: 70 (4); Resistance: 70 (5); Concurrent: 70 (5)	Aerobic: 35.9 (4.4); Resistance: 36.7 (5.8); Concurrent: 35.8 (4.5)	12/28	70 (5)	36.7 (5.0)	Aerobic: 3 d/wk 60–90 min/session; Treadmill 65%HR, then to 70–85%HR– Resistance: 1–2 sets of 8–12 reps at 65%1RM and progressed to 2–3 sets at 85%1RM; 9 exercises total	6 months	MRI	Nutritional counseling to achieve a 500–750 kcal/d deficit in energy requirements and 1 g protein/kg body weight/d
Meir et al., 2016	Abdominal Obesity	Thigh InterMAT	Low fat: 38; Low carbohydrate/Mediterranean: 40	All: 47.9 (9.3)	All: 30.87 (3.84)	Low fat control 37; Low carbohydrate/Mediterranean control: 35	All: 47.9 (9.3)	All: 30.87 (3.84)	3x/week; Aerobic: 20 min at 65% HR first month, then 60 min at 80% HR the rest; Resistance: 1 set at 60%1RM first month, then 2 sets at 80%1RM with multiple exercises	12 months	MRI	Diets aimed at an energy intake of 1,500 kcal/day for women and 1,800 kcal/day for men, with restricted intake of trans fats and refined carbohydrates and increased intake of vegetables. Lunch, which is typically the main meal in this population, was provided exclusively by the workplace cafeteria during the work week. A dietician worked closely with the kitchen staff to adjust the diets to the specific diet groups. The 18-mo dietary intervention included a weekly 90-min nutritional session with clinical dieticians in the workplace during the first month of intervention, and monthly meetings thereafter. To maintain equal intensity of treatment, the workshop format and the quality of the materials were similar across the diet groups, except that the instructions and materials were specific to each diet strategy (low carbohydrate & Mediterranean or low-fat diet).
Shea et al., 2011	Overweight & Obesity	Thigh InterMAT	12/10	Women: 71.3 (4.9); Men: 69.5 (3.9)	Women: 31.1 (3.1); Men: 33.2 (5.1)	12/10	Women: 70.0 (2.6); Men: 70.5 (4.7)	Women: 33.8 (5.4); Men: 31.5 (2.8)	3x/week; week 1—two sets of 8–10 reps at 40–50% of one RM; week 2—three sets of 8–10 reps at 50–60% of one RM; weeks 3–16—three sets of 8–10 reps at 70% of one RM. Participants rested for ~ 2–3 min between each set	16 weeks	CT	Each participant met with a study dietitian and were assigned a meal plan to provide a daily energy deficit of 500 kcal calculated from estimated total daily energy requirements for weight maintenance, using formulas established by the Institute of Medicine and the National Academy of Science. Two meal replacements per day (bars and shakes) were provided to all participants (containing ~ 220 kcal with 7–10 g protein, 33–46 g carbohydrates, and 1.5–5 g fat with 2–5 g of fiber). For the third meal, a weekly menu plan with recipes was given to the participants. This meal was composed of traditional foods, was low in fat, high in vegetables but allowed for individual preferences, and provided 500–750 kcal. In addition, up to three snacks (~ 100 kcal each) were allowed each day. The macronutrient goal for each individual was ~ 55–60% carbohydrates, ~ 15% protein, and ~ 25% fat. All participants attended weekly group behavioral and educational sessions. The sessions (n = ~ 10 per group) were conducted by a registered dietitian with expertise working with older adults on diet behavior modification. At each weekly session, all participants turned in food diaries which they were asked to complete daily for self-monitoring, and body weight was measured and recorded.
Brubaker et al., 2023	HFpEF & Obesity	Thigh IntraMAT	6/38	69.7 (5.8)	39.2 (5.6)	7/37	67.9 (5.4)	40.0 (5.9)	3x/wk (40 min aerobic; 20 min resistance); stationary cycling and 8–12 reps of 1 set 20–30%1RM first 4 weeks; 2 sets at 40–50%1RM after week 4; 12 sets in total of resistance exercises	20 weeks	MRI	Hypocaloric diet using meals (lunch, dinner, and snacks) prepared under the direction of a registered dietitian; No participant received less than 1100 and 1300 kcal/day for female and male participants, respectively. For safety, total weight loss was capped and did not exceed > 15% of body weight or a BMI of < 25 kg/m^2^. The tailored meals provided a balanced diet with < 30% calories from fat and at least 1.2 g protein/kg body weight/d
Gorgey et al., 2012	Spinal Cord Injury	Thigh IntraMAT	5/0	36 (9)	21 (5)	4/0	33 (10)	23 (3)	2x/week; 4 sets of 10 reps; additional 2 lbs workload weekly; 2 min rest between sets	12 weeks	MRI	All participants were asked to follow a standard diet (45% CHO, 30% fat, and 25% protein) protocol during the study (9). Food diaries were recorded daily for the duration of 12-wk study and were analyzed weekly using the Nutrition Data System Software for Research Program (http://www.ncc.umn.edu) by a dietitian to assess the caloric intake. The software is designed to determine the caloric intake and the relative contribution of CHO, protein, and fat intakes. Foods consumed were self-selected, and no supplements were provided. To ensure adherence to the standard diet program, weekly feedback was provided by a dietitian through interview, telephone calls, or e-mails.
Ryan et al., 2014	Obesity	Erector spinae, Lateral and rectus abdominal, and Psoas IntraMAT	0/43	All: 50–76	32 (7.6)	0/22	All: 50–76	34 (5.7)	3x/wk; treadmill and elliptical trainers at 50–60% and gradually up to > 85%HR for 45 min	6 months	CT	A registered dietitian was consulted for 1 day/week for 6 − 8 weeks in order to maintain the dietary composition across the study: the subjects were already “weight stable” on the diet at baseline testing and were instructed to maintain their diet throughout the study. After the baseline testing, the women attended weekly weight-loss classes for 6 months for diet instructions led by the registered dietitian. Compliance with the diet was monitored using 7-day food records developed by the American Diabetes Association as part of the Exchange List System. To generate a caloric deficit, the dietitian estimated the subjects’ total energy expenditure (TEE). To induce weight loss, 350 − 500 kcal was subtracted from the TEE.
Christiansen et al., 2009	Obesity	L2/L3 level InterMAT	10/11	37.5 (8)	34.2 (3)	10/9	35.6 (7)	35.3 (4)	3x/week; Duration of 60–75 min per training session, with an estimated energy expenditure of 500–600 kcal per session. The subjects could choose between different modes of exercise; stationary bicycling, jogging on a treadmill or stair stepping. The Karvonen method for exercise intensity was used to target an exercise intensity of 70% HR	12 weeks	MRI	Low energy diet of 600–800 kcal/d for 8 weeks; maintained for 4 weeks. To ensure compliance to the diet, subjects in both groups were allowed to consume ad libitum low-energy vegetables and were followed every second week by clinical staff 55% from carbohydrates, 15% from protein, and 30% from fat. All subjects in the three groups were asked to keep dietary intake records over a 2-week period.
Janssen et al., 2002	Obesity	Abdominal InterMAT	Aerobic: 0/11; Resistance: 0/14	Aerobic: 37.5 (6); Resistance: 34.8 (5.8)	Aerobic: 36 (7.1); Resistance: 31.6 (4.3)	0/13	40.1 (6.7)	33.7 (4.1)	Aerobic: 5x/wk; exercise sessions lasted 15 min at the beginning and progressed to a maximum of 60 min based on the subject’s capabilities. The mode of aerobic exercise was determined by the subject and consisted of either walking on a motorized treadmill and progressed from 50 to 85% of the maximal heart rate that was achieved during the maximal oxygen uptake test– Resistance: 3d/wk; Seven exercises were performed in each session: leg extension, leg flexion, super pullover (latissimus dorsi), bench press, shoulder press, triceps extension, and biceps curl. One set of 8–12 repetitions were performed to the point of volitional fatigue	16 weeks	MRI	All subjects were required to keep daily diet records for the duration of the study and to limit their dietary fat intake to 30%. The diet records were reviewed using standard food tables. All subjects attended weekly meetings to obtain dietary counsel and discuss success strategies. After the 16-week treatment period, the energy intake for weight maintenance was recalculated and prescribed until completion of the post-test measurements.

BMI, body mass index; CT, computed tomography; HFpEF, heart failure with preserved ejection fraction; HR, heart rate; InterMAT, intermuscular adipose tissue; IntraMAT, intramuscular adipose tissue; RM, repetition maximum; MRI, magnetic resonance imaging

**Table 2:** Study and participant characteristics of the included studies without a hypocaloric dietary protocol. Data are expressed as mean (standard deviation) or interquartile range

Study, year	Health status	Outcome of interest	Intervention			Control			Training protocol	Duration	Assessment tool	Dietary intake control
			*n* (M/F)	Age (SD)	BMI (kg/m^2^)	*n* (M/F)	Age (SD)	BMI (kg/m^2^)				
Jung et al., 2012	Overweight & T2D	Thigh IntraMAT	Vigorous: 0/8; Moderate: 0/8	Vigorous: 48.4 (6.1); Moderate: 56.8 (8.2)	Vigorous: 25.9 (1.6); Moderate: 25.5 (1.5)	0/12	55.5 (7.6)	27.7 (3.4)	Aerobic exercise for 5x/wk; >7 METs for 60 min (vigorous intensity) − 5x/wk; 3.5–5.2 METs moderate intensity for 30 min	12 weeks	CT	Baseline and every 2 weeks for 12 weeks; Energy intake surveys were filled out for 3 days in the form of subject daily food intake records (2 weekdays and 1 weekend day).
Ku et al., 2010	T2D	Thigh IntraMAT	Aerobic: 0/5; Resistance: 0/13	Aerobic: 55.7 (7); Resistance: 55.7 (6.2)	Aerobic: 27.1 (2.4); Resistance: 27.1 (2.3)	0/16	57.8 (8.1)	27.5 (2.8)	Aerobic: walking 60 min at moderate intensity (3.6–5.2 METs)– Resistance: 5d/wk 40–50%1RM for 15–20 reps and 3 sets; elastic band exercises	12 weeks	CT	Every 2 weeks for 12 weeks; All participants received information about how to monitor their diet and exercise. Daily activities of all the participants were monitored with a Lifecorder^®^ (Suzuken Co., Nagoya, Japan) and dietary intake was selfreported, using a 3-day diet diary.
Murphy et al., 2012	Community-dwelling	Thigh InterMAT	6/10	59 (5.1)	26.8 (4.9)	2/4	55.7 (5.2)	27.2 (4.8)	6x/wk for 60 min/session at 71% HR (walking, elliptical exercise, cycling, running)	1 year	MRI	The participants kept diaries of their food intake on a regular basis during the study; information from the diaries was used by the dietitians as the basis for dietary counseling and for subjective monitoring of the subjects’ progress re weight loss
Ogawa et al., 2020	Healthy	Thigh InterMAT and IntraMAT	13/0	31.3 (7.6)	24.1 (1.9)	7/0	34 (7.1)	24.6 (2.5)	3x/wk; 8–10 reps lower body at 80%1RM	8 weeks	MRI	Daily meals during bed rest were provided according to the German Nutrition Society guidelines (50–55% carbohydrate, 30–35% fat, and 15–20% protein)
Fortuin-de Smidt et al., 2020	Obesity	Soleus and Tibialis anterior InterMAT	0/23	22 (21–24)	34.1 (2.8)	0/22	23 (21–27)	33.4 (2.7)	Aerobic: 4x/wk; 40–60 min moderate/vigorous intensity 75–85%HR and 60–70% HRR of strength exercises	12 weeks	MRI	Dietary intake was recorded at baseline, weeks 4, 8, and 12
Keating et al., 2017	Obesity	L4/L5 Psoas InterMAT	2/13	45.4 (5.8)	32.2 (5.1)	2/12	44.2 (6.5)	30.8 (4.7)	2-3x/wk; 2–3 sets 8–12 reps up to 80%1RM	8 weeks	MRI	Baseline and Post-Intervention; Three-day diet diaries (1 weekend day, 2 weekdays) were obtained during week 1 and week 8 of the study and analyzed by a dietitian blinded to group allocation.
Minett et al., 2020	Community-dwelling	Distal tibia IntraMAT	19/25	77.1 (74.3–84.1)	Height: 163.8 (155.5–172.8) cm; Weight: 71.3 (60.9–83.6) kg	18/23	75.8 (73.8–80.1	Height: 167.5 (15.5–174.3) cm; Weight: 76.6 (66.1–90.3) kg	5x/wk; 30 min of walking for 2 days and Esslinger Program for 3 days	12 weeks	pQCT	Baseline and Post-Intervention; Used 24-h diet recall records and the Three Factor Eating Questionnaire (TFEQ) to note changes in diet and eating habits. Measurements were made at baseline and 3 months.

BMI, body mass index; CT, computed tomography; HR, heart rate; InterMAT, intermuscular adipose tissue; IntraMAT, intramuscular adipose tissue; RM, repetition maximum; MET, metabolic equivalent of task; MRI, magnetic resonance imaging; pQCT, Peripheral quantitative computed tomography; T2D, type 2 diabetes

Tables S3 and S4 show a descriptive summary of the results for each type of exercise on compartments of InterMAT and IntraMAT.

### Effects of resistance exercise vs. control on thigh InterMAT with a hypocaloric diet

Following resistance exercise and a hypocaloric diet, a benefit was displayed in terms of thigh InterMAT in adults with overweight and obesity after 10 weeks vs. control (resistance exercise ◊ Δ = -1.5 (0.5) cm^2^, *p* < 0.01; control ◊ Δ = -0.9 (0.4) cm^2^, *p* = 0.07) [48]. Similarly, but in the longer term (6 months) the study by Waters et al., (2022) also showed a benefit on thigh InterMAT following resistance exercise (Δ = -101 (19.9) cm^3^, *p* < 0.05) vs. control (Δ = -9 (20) cm^3^) (*p* < 0.01) [15]. On the contrary, no statistical differences were observed after 16 weeks of resistance exercise in men and women with overweight and obesity, separately [49] (Men ◊ resistance exercise: Δ = -6.4 (3.3) cm^3^/5 cm vs. control: Δ = -3.4 (3.4) cm^3^/5 cm; Women ◊ resistance exercise: Δ = -8.9 (2.7) cm^3^/5 cm vs. control: Δ = -12.1 (2.8) cm^3^/5 cm). More recent research employing a longer-term protocol (18 months) in adults with obesity also demonstrated identical results by Madrid et al., (2023) (resistance exercise: Δ = -2.55 (2.94) cm^2^; control: Δ = -2.43 (3.07) cm^2^) [32]. Lastly, a protocol employing resistance training for 5 months in adults with overweight and obesity showed a significant reduction of thigh InterMAT in both the resistance exercise and control groups, however, without any between-group differences (resistance exercise: Δ = -3.8 (6.8) cm^3^; control: Δ = -2.2 (6.2) cm^3^) (*p* > 0.05) [33].

### Effects of resistance exercise on InterMAT and thigh InterMAT vs. control with a hypocaloric diet

Only one study measured InterMAT in another tissue and thigh IntraMAT following resistance exercise. Janssen et al., (2002) found a significant difference after 16 weeks in adults with obesity, but without between-group changes (resistance exercise: Δ = -0.12 (0.14) kg; control: Δ = -0.22 (0.19), *p* < 0.05) [37]. Regarding thigh IntraMAT, a significant decrease was shown in the resistance training vs. control (*p* < 0.01) after 12 weeks in individuals with spinal cord injury [38].

### Effects of resistance exercise vs. control on InterMAT without a hypocaloric diet

During protocols that did not employ a hypocaloric diet, no statistically significant changes were shown with resistance exercise alone on thigh InterMAT in healthy individuals after 8 weeks (pre-intervention: 427.4 ± 219.3 cm^3^ to post-intervention: 432.2 ± 206.8 cm^3^ vs. pre-control: 375.0 ± 128.4 cm^3^ to post-control: 355.5 ± 182.4 cm^3^; *p* > 0.05) [17]. This was also demonstrated in adults with type 2 diabetes following 12 weeks of resistance exercise (Δ = 4 (199) g) vs. control (Δ = -32 (171) g) (*p* > 0.05) [50]. When L4/L5 psoas InterMAT was assessed, identical results were observed after 8 weeks in adults with obesity (resistance exercise ◊ pre: 122 (13) cm^3^ to post: 115 (13.5) cm^3^; control ◊ pre: 126 (21) cm^3^ to post: 142 (23) cm^3^) [45].

### Effects of aerobic exercise vs. control on thigh intramat and thigh InterMAT with or without a hypocaloric diet

In terms of aerobic exercise, no InterMAT differences were found after 18 months in adults with obesity (Δ = -2.89 (2.92) cm^2^) vs. control (Δ = -2.43 (3.07) cm^2^) [32]. Conversely, another study conducted in adults with obesity for 6 months showed a significant reduction in thigh InterMAT (Δ = -105 (18.9) cm^3^) vs. control (Δ = -9 (20) cm^3^) (*p* < 0.01) [15].

When a hypocaloric diet was absent, Ku et al., (2010) found no changes from aerobic exercise (Δ = -31 (159) g) vs. control (Δ = -32 (171) g) after 12 weeks regarding thigh IntraMAT [50]. Moreover, in relation to thigh InterMAT, the study from Murphy et al., (2012) [51] demonstrated a statistically significant reduction following aerobic exercise vs. control in the absence of a hypocaloric diet after 12 months (Δ = -45 ± 8.16 ml vs. -25 ± 7.45 ml; *p* < 0.05). However, these changes were not observed following vigorous or moderate intensity of aerobic exercise (*p* > 0.05) for 12 weeks [52].

### Effects of aerobic exercise vs. control on InterMAT with or without a hypocaloric diet

InterMAT of psoas at L2/L3 level and the abdomen following aerobic exercise after 6 months in individuals with obesity was not reduced following a hypocaloric protocol vs. control (*p* > 0.05 between groups) [38]. Moreover, IntraMAT of erector spinae, lateral and rectus abdominal, and psoas at L4/L5 level following aerobic exercise with a hypocaloric diet did not induce any significant changes between groups (*p* > 0.05) [53]. Likewise, similar findings were found regarding IntraMAT of the distal tibia following combined aerobic with resistance exercise, but without a hypocaloric diet (*p* > 0.05). Additionally, another study examining psoas InterMAT at L2/L3 level did not show a difference after 12 weeks in adults with obesity (aerobic exercise: Δ = -8-11%; control: Δ = -7%) [39]. Finally, a significant reduction in abdominal InterMAT was shown after 16 weeks in adults with obesity in both aerobic and control groups, however, a between group analysis was not conducted (aerobic exercise: Δ = -38 (0.4) kg; control: Δ = -22 (0.19), *p* < 0.05) [37].

### Effects of concurrent exercise vs. control on thigh InterMAT and thigh InterMAT with or without a hypocaloric diet

When resistance and aerobic exercise was combined with a hypocaloric diet, no changes were observed in adults with obesity after 6 months in regards to thigh InterMAT (concurrent exercise: Δ = -0.07 (0.07) kg; control: Δ = 0.0 (0.05) kg)) [54]. No changes in thigh InterMAT were also presented after a hypocaloric diet from a low fat or low carbohydrate diet al.ongside concurrent exercise in the CENTRAL trial [55]. Lastly, in adults with heart failure and preserved ejection fraction with obesity, no changes were revealed after 20 weeks of concurrent exercise on thigh IntraMAT with a hypocaloric diet (Δ = -4 (3.2) cm^2^) vs. control (Δ = − 4 (3.2) cm^2^) [56].

Similarly, when a hypocaloric diet was not followed in adults with obesity for 12 weeks, InterMAT from soleus and tibialis anterior, and psoas at L4/L5 level, were not reduced following a combined aerobic with resistance exercise vs. an resistance exercise regime alone, respectively [44]. Minett et al., (2020) also investigated the impact of concurrent exercise for 12 weeks in community-dwelling individuals, however, no changes were also displayed in terms of distal tibia IntraMAT between groups (concurrent exercise: Δ = -39.6 (65.9) mm^2^; control: Δ = -94.7 (71.6) mm^2^, *p* < 0.05) [46].

### Risk of bias assessment

From the included studies for which a hypocaloric diet was introduced, three had an overall low risk of bias [19, 30, 37], seven had some concerns [31, 33–35, 38–40], and two had an increased risk of bias [32, 36] (Table S5). In addition, from the studies that did not introduce a hypocaloric diet, one study had an overall low risk of bias [41], four had some concerns [43–45, 47], and two had a high risk of bias [42, 46] (Table S6).

## Discussion

In this systematic review, we explored the impact of exercise with or without a hypocaloric diet on InterMAT and IntraMAT through different compartments. Albeit exercise, either aerobic, resistance, or concurrent, may offer multiple health-related benefits, results were inconsistent following a hypocaloric diet compared to control, while no changes were demonstrated when a hypocaloric diet was not followed.

Recently, a previous meta-analysis found a small effect of exercise (mean *g* = 0.21, *p* = 0.03) on muscle quality [21], in which, both InterMAT and IntraMAT were included in its definition. It was unknown, however, whether different types of exercise would exhibit differing results, considering that the relationship of skeletal muscle metabolism with aerobic and resistance exercise may differ substantially. Similarly, another meta-analysis’ pooled effect estimates from three studies that utilized different types of exercise, showed identical results (SMD: 0.45, 95% CI: 0.05–0.86) [22]. Similar findings pertinent to effect estimate were displayed in another study, which also mixed exercise types and was prone to double-counting, including in the same analysis two or more control groups of the same cohort [24]. Lastly, the study by Ramírez-Velez et al. (2021) [23] followed a similar methodological protocol, demonstrating significant reductions due to exercise; however, the inclusion of studies measuring muscle attenuation was also made. It is worth noting, that none of the previous meta-analyses had inclusion criteria in terms of collecting studies that would control, to an extent, for dietary intake and a hypocaloric diet, which are critical confounders.

Our narrative synthesis found no conclusive evidence regarding the impact of different exercise types with or without a hypocaloric diet on thigh InterMAT and IntraMAT, as well as InterMAT and IntraMAT from other compartments. Some of these findings are reflected by the lack of available studies that could reveal more reliable conclusions, especially in allowing to perform a meta-analysis. Interestingly, the majority of studies examined thigh InterMAT with a hypocaloric diet, for which, although statistical findings between groups were presented for both aerobic and resistance exercise, other studies with homogeneous populations and study durations found no differences vs. control. The studies that examined concurrent exercise, even in the presence of hypocaloric diet, did not demonstrated any reductions in thigh InterMAT and IntraMAT. Therefore, albeit trials controlling for dietary intake while employing a hypocaloric diet during exercise may have potential in reducing fat infiltration in and around the (thigh) skeletal muscle, more research is warranted. When a non-hypocaloric diet was followed, exercise of any type did not confer any benefits overall.

### Clinical and statistical implications

The results of this systematic review have significant clinical and practical implications. Metabolic diseases such as obesity and diabetes have reached very high prevalence rates globally, and one of the ways to address these conditions is through lifestyle changes, such as diet and exercise. Often, these specific interventions are implemented as monotherapies rather than combined interventions within a more holistic approach. Our results indicated that it may be important to combine exercise with a hypocaloric diet to reduce adipose tissue levels in skeletal muscle, albeit more research is needed to confirm the intensity of exercise and magnitude of caloric deficit and/or macronutrient composition. Hence, physicians, exercise scientists, and dietitians should collaborate harmoniously to provide targeted interventions, as this appears to be the most effective approach, ensuring a negative energy balance.

### Strengths and limitations

This is the first systematic review that examined different types of exercise in InterMAT and IntraMAT separately, including studies that attempted to control for dietary intake, with or without a hypocaloric diet. By applying these criteria, we sought to carefully examine the effect of different types of exercise on fat infiltration in and around different muscle tissues and provide further insights to clinicians and researchers in exploring ways to improve skeletal muscle and overall metabolic health. However, our study was prone to several limitations. First, considering the low number of studies by applying such inclusion criteria, we were not able to explore the impact of age, sex, different health status, and assessment tools on our findings. Moreover, we could not make conclusions based on the intensity, frequency, and duration of the exercise protocol used among studies, while in terms of caloric intake, we could not account for the magnitude of calorie deficit that was varied significantly. Finally, the different methods used to control for dietary intake, including nutritional education, frequency of food diary collection, and overall methods of nutritional assessment, among studies is another limitation.

## Conclusion

This systematic review investigated the effects of different exercise types, both with and without a hypocaloric diet, on InterMAT and IntraMAT in various muscle compartments. Although some studies suggest potential benefits from combining aerobic and/or resistance exercises with a hypocaloric diet, no statistically significant differences were observed compared to control when exercise overall was performed without a caloric deficit. These findings may be influenced by the considerable variability in exercise protocols and dietary interventions, as well as the limited number of studies available. Therefore, more research is needed to assess the impact of exercise type, intensity, and dietary control on fat infiltration in skeletal muscle under hypocaloric conditions.

## Electronic supplementary material

Below is the link to the electronic supplementary material.

Supplementary Material 1

Supplementary Material 2

Supplementary Material 3

Supplementary Material 4

Supplementary Material 5

Supplementary Material 6

## Data Availability

No datasets were generated or analysed during the current study.
