# Supplementary material for: Effects of exercise with or without a hypocaloric diet on intermuscular and intramuscular fat: a systematic review
Source: Aging Clin Exp Res. 2025 Jun 9;37(1):183. doi: 10.1007/s40520-025-03097-2 (PMC12149019; doi:10.1007/s40520-025-03097-2)
Supplement: Supplementary file 1 — Supplementary Material 1 [file 40520_2025_3097_MOESM1_ESM.docx]

**Table S1.**Search terms employed in the screening based on title, abstract, and keywords in the literature search.

| **Database** | **Search terms** |
| --- | --- |
|  |  |
| PubMed | (strength training OR resistance exercise OR resistance training OR aerobic exercise OR aerobic training OR concurrent training  OR concurrent exercise OR high-intensity interval training OR “HIIT exercise” OR circuit training OR circuit exercise OR  physical activity) AND (“muscle quality” OR “muscle attenuation” OR "adipose infiltration" OR "myosteatosis" OR "skeletal muscle fat"  OR "muscle fat fraction" OR “muscle fat deposition” OR "intramuscular fat" OR "intermuscular fat" OR "intramuscular adipose tissue"  OR "intermuscular adipose tissue" OR "intramuscular adiposity" OR "intermuscular adiposity" OR "fat infiltration" OR  “fatty infiltration”) |
| Cochrane Library | (strength training OR resistance exercise OR resistance training OR aerobic exercise OR aerobic training OR concurrent training  OR concurrent exercise OR high-intensity interval training OR “HIIT exercise” OR circuit training OR circuit exercise OR  physical activity) AND (“muscle quality” OR “muscle attenuation” OR "adipose infiltration" OR "myosteatosis" OR "skeletal muscle fat"  OR "muscle fat fraction" OR “muscle fat deposition” OR "intramuscular fat" OR "intermuscular fat" OR "intramuscular adipose tissue"  OR "intermuscular adipose tissue" OR "intramuscular adiposity" OR "intermuscular adiposity" OR "fat infiltration" OR  “fatty infiltration”) |
| Web of Science | (strength training OR resistance exercise OR resistance training OR aerobic exercise OR aerobic training OR concurrent training  OR concurrent exercise OR high-intensity interval training OR “HIIT exercise” OR circuit training OR circuit exercise OR  physical activity) AND (“muscle quality” OR “muscle attenuation” OR "adipose infiltration" OR "myosteatosis" OR "skeletal muscle fat"  OR "muscle fat fraction" OR “muscle fat deposition” OR "intramuscular fat" OR "intermuscular fat" OR "intramuscular adipose tissue"  OR "intermuscular adipose tissue" OR "intramuscular adiposity" OR "intermuscular adiposity" OR "fat infiltration" OR  “fatty infiltration”) |
| Scopus | ( resistance AND exercise OR aerobic AND exercise OR concurrent AND training OR concurrent AND exercise OR circuit AND training OR circuit AND exercise ) AND ( "muscle quality" OR "muscle attenuation" OR "adipose infiltration" OR "myosteatosis" OR "skeletal muscle fat" OR "muscle fat fraction" OR "muscle fat deposition" OR "intramuscular fat" OR "intermuscular fat" OR "intramuscular adipose tissue" OR "intermuscular adipose tissue" OR "intramuscular adiposity" OR "intermuscular adiposity" OR "fat infiltration" OR "fatty infiltration" ) |
