# Supplementary material for: Effects of exercise with or without a hypocaloric diet on intermuscular and intramuscular fat: a systematic review
Source: Aging Clin Exp Res. 2025 Jun 9;37(1):183. doi: 10.1007/s40520-025-03097-2 (PMC12149019; doi:10.1007/s40520-025-03097-2)
Supplement: Supplementary file 2 — Supplementary Material 2 [file 40520_2025_3097_MOESM2_ESM.docx]

**Table S2.** PICOS (Population, Intervention, Comparison, Outcome, and Study Design) principles.

|  | Items | Details |
| --- | --- | --- |
| Participants | Adults aged > 18 years irrespective of health status |  |
| Intervention | Exercise of any type with or without a hypocaloric controlled diet | Aerobic, exercise, or concurrent of any intensity and frequency. Additionally, the studies had to follow some type of dietary intake control during and/or around the study (i.e., baseline and during follow-up), or participants were provided a dietary supervision from authors or a healthcare professional, such as a registered nutritionist or dietitian |
| Comparison | A hypocaloric or non-hypocaloric diet without exercise |  |
| Outcomes | Intramuscular and intermuscular fat from any body compartment |  |
| Study design | Randomised controlled trials |  |
