# Supplementary material for: Effects of exercise with or without a hypocaloric diet on intermuscular and intramuscular fat: a systematic review
Source: Aging Clin Exp Res. 2025 Jun 9;37(1):183. doi: 10.1007/s40520-025-03097-2 (PMC12149019; doi:10.1007/s40520-025-03097-2)
Supplement: Supplementary file 3 — Supplementary Material 3 [file 40520_2025_3097_MOESM3_ESM.docx]

**Table S3.** Effects of exercise on IntraMAT or InterMAT of other muscle compartments following a hypocaloric protocol.

| Study | Type of exercise | Compartment | Baseline-intervention | Post-intervention | Baseline-control | Post-control |
| --- | --- | --- | --- | --- | --- | --- |
| Brubaker 2023 | Concurrent | Thigh IntraIMAT | 32±10 cm^2^ | Δ = –4 (–5, –3) cm^2^ | 34±11 cm^2^ | Δ = –4 (–5, –3) cm^2^ |
| Gorgey 2012 | Resistance | Thigh IntraIMAT | 18±10% | 15±8%, p = 0.06 | 28±25% | 31±24%, p = 0.08 |
| Ryan 2014 | Aerobic | Erector spinae, Lateral and rectus abdominal, and Psoas IntraIMAT | Erector spinae: 11.9±6.96; Psoas: 4.4±6.76; Lateral abdominal area: 11.4±7.16; Rectus abdominis: 3.2±6.76 | Erector spinae: 11.2±6.98; Psoas:  4.0± 6.68;  Lateral abdominal area: 10.3±7.08; Rectus abdominis: 2.9±6.68;  All p <0.05 or <0.01 | Erector spinae: 11.9±5.69 SD; Psoas: 4.5±5.09; Lateral abdominal area: 12.7±5.69; Rectus abdominis: 3.6±5.09 | Erector spinae:  11.1± 5.49;  Psoas: 4.0±4.89; Lateral abdominal area: 11.0±5.49; Rectus abdominis: 3.5±5.09; Only Lateral Abdominal Area had p < 0.05 |
| Christiansen 2009 | Aerobic | L2/L3 level InterIMAT | - | Δ = -8-11% (p = 0.06 vs. diet only group) | - | Δ = -7% |
| Janssen 2002 | Aerobic | Abdominal InterIMAT | - | Δ = -0.38 (0.4) kg; p < 0.05 | - | Δ = -0.22 (0.19) kg; p < 0.05 |
| Janssen 2002 | Resistance | Abdominal InterIMAT | - | Δ = -0.12 (0.14) kg; p < 0.05 | - | Δ = -0.22 (0.19) kg; p < 0.05 |

Data are reported as mean (±SD) or 95%CI.
