# Supplementary material for: Effects of exercise with or without a hypocaloric diet on intermuscular and intramuscular fat: a systematic review
Source: Aging Clin Exp Res. 2025 Jun 9;37(1):183. doi: 10.1007/s40520-025-03097-2 (PMC12149019; doi:10.1007/s40520-025-03097-2)
Supplement: Supplementary file 4 — Supplementary Material 4 [file 40520_2025_3097_MOESM4_ESM.docx]

**Table S4.** Effects of exercise on IntraMAT or InterMAT of other muscle compartments without a hypocaloric protocol.

| Study | Type of exercise | Compartment | Baseline-intervention | Post-intervention | Baseline-control | Post-control |
| --- | --- | --- | --- | --- | --- | --- |
| Murphy 2012 | Aerobic | Thigh InterMAT | 96 (13.36) ml | Δ = -45 (8.16) ml | 77 (16) ml | Δ = -25 (7.45) ml |
| Ogawa 2020 | Resistance | Thigh InterMAT | 427.4 ± 219.3 cm^3^ | 432.2 ± 206.8 cm^3^ | 375.0 ± 128.4 cm^3^ | 355.5 ± 182.4 cm^3^ |
| Ogawa 2020 | Resistance | Thigh IntraMAT | 17.1 ± 3.1% | Δ = −2.1 (−3.7, −0.5)% | 17.3 ± 1.1% | Δ = −0.3 (−2.4, 1.8)% |
| Ku 2010 | Resistance | Thigh IntraMAT | 412 ± 160 g | Δ= 4 ± 199 g | 564 ± 222 g | Δ = −32 ± 171 g |
| Fortuin-de Smidt 2020 | Concurrent | Soleus and Tibialis anterior InterMAT | Soleus:  10.25  (9.50-11.50)%;  Tibialis anterior: 3.89%  (3.20-5.60)% | Soleus:  9.6 (8.00-10.90)% or  Δ = −6.34%;  Tibialis anterior: 3.79 (3.10-5.10)% or Δ = −2.57% | Soleus: 10.17 (7.40-13.44)%; Tibialis anterior: 4.09 (2.80-5.60)% | Soleus: 9.67 (8.44-11.94)% or  Δ = -4.92%;  Tibialis anterior: 4.19 (1.50-5.09)% or Δ = 2.44% |
| Keating 2017 | Resistance | L4/L5 level Psoas InterMAT | 122 (16.87) cm^3^ | 115.0 (13.54) cm^3^ | 126 (20.96) cm^3^ | 142 (23.04) cm^3^ |
| Minett 2020 | Concurrent | Distal tibia IntraMAT | 494 (282–716) mm^2^ | Δ = -39.61 (65.87) mm^2^ | 529 (364–744) mm^2^ | Δ = -94.66 (71.64) mm^2^ |

Data are reported as mean (±SD) or 95%CI.
