# Supplementary material for: Effects of exercise with or without a hypocaloric diet on intermuscular and intramuscular fat: a systematic review
Source: Aging Clin Exp Res. 2025 Jun 9;37(1):183. doi: 10.1007/s40520-025-03097-2 (PMC12149019; doi:10.1007/s40520-025-03097-2)

**Table S5.** Included studies exploring the impact of exercise on intermuscular and intramuscular fat with a hypocaloric diet.


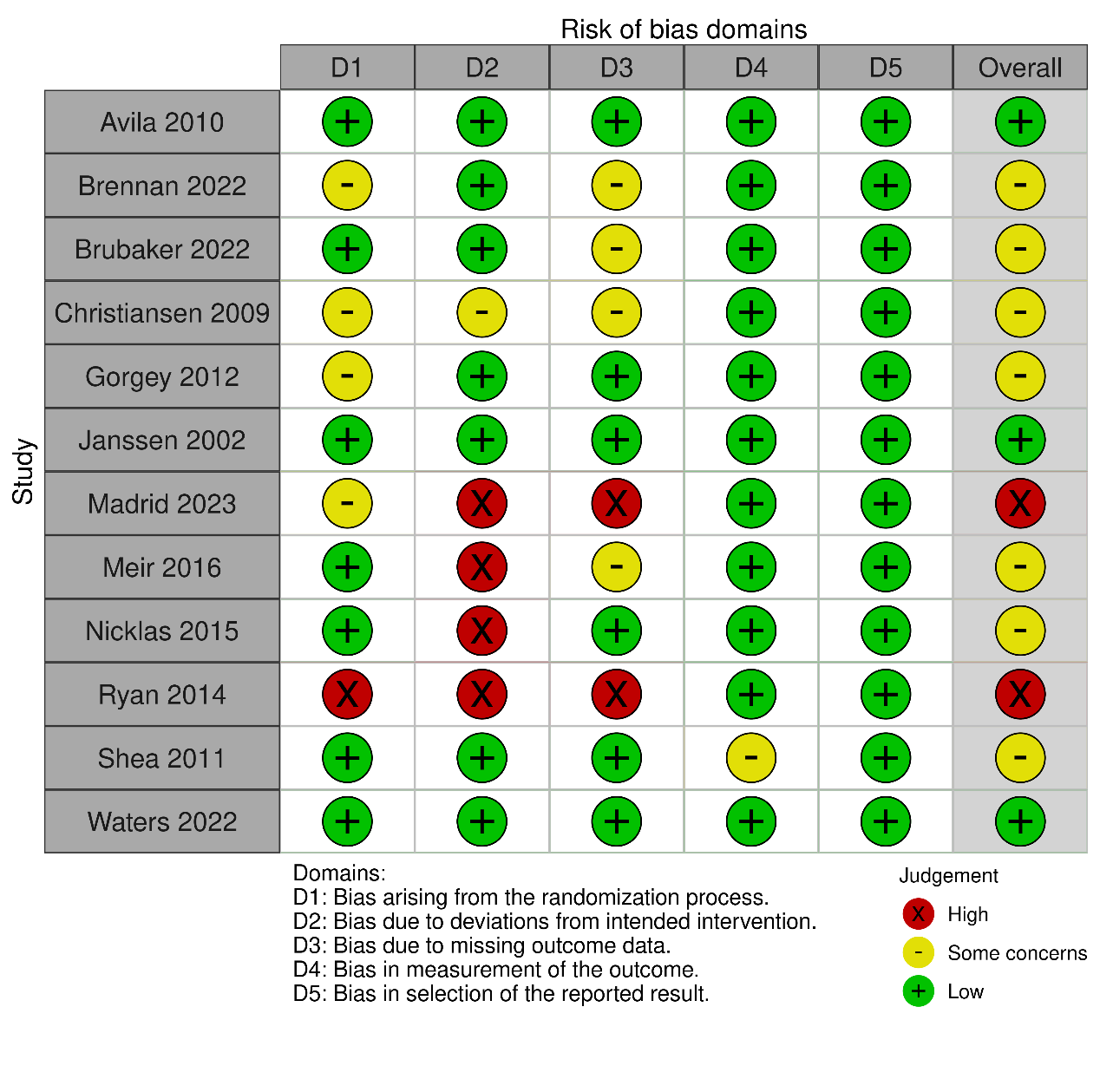

Supplement: Supplementary file 5 — Supplementary Material 5 [file 40520_2025_3097_MOESM5_ESM.docx]
